# Mechanoregulation of YAP and TAZ in Cellular Homeostasis and Disease Progression

**DOI:** 10.3389/fcell.2021.673599

**Published:** 2021-05-24

**Authors:** Xiaomin Cai, Kuei-Chun Wang, Zhipeng Meng

**Affiliations:** ^1^Department of Oncology, The First Affiliated Hospital of Nanjing Medical University, Nanjing, China; ^2^School of Biological and Health Systems Engineering, Arizona State University, Tempe, AZ, United States; ^3^Department of Molecular and Cellular Pharmacology, University of Miami Miller School of Medicine, Miami, FL, United States; ^4^Sylvester Comprehensive Cancer Center, University of Miami Miller School of Medicine, Miami, FL, United States

**Keywords:** YAP, TAZ, the Hippo pathway, mechanotransduction, ECM stiffness, stretch, flow shear, contact inhibition of cells

## Abstract

Biophysical cues, such as mechanical properties, play a critical role in tissue growth and homeostasis. During organ development and tissue injury repair, compressive and tensional forces generated by cell-extracellular matrix or cell-cell interaction are key factors for cell fate determination. In the vascular system, hemodynamic forces, shear stress, and cyclic stretch modulate vascular cell phenotypes and susceptibility to atherosclerosis. Despite that emerging efforts have been made to investigate how mechanotransduction is involved in tuning cell and tissue functions in various contexts, the regulatory mechanisms remain largely unknown. One of the challenges is to understand the signaling cascades that transmit mechanical cues from the plasma membrane to the cytoplasm and then to the nuclei to generate mechanoresponsive transcriptomes. YAP and its homolog TAZ, the Hippo pathway effectors, have been identified as key mechanotransducers that sense mechanical stimuli and relay the signals to control transcriptional programs for cell proliferation, differentiation, and transformation. However, the upstream mechanosensors for YAP/TAZ signaling and downstream transcriptome responses following YAP/TAZ activation or repression have not been well characterized. Moreover, the mechanoregulation of YAP/TAZ in literature is highly context-dependent. In this review, we summarize the biomechanical cues in the tissue microenvironment and provide an update on the roles of YAP/TAZ in mechanotransduction in various physiological and pathological conditions.

## Introduction

The fate of individual cells is shaped and determined by both biochemical and biophysical factors in cellular microenvironments (Discher et al., 2005; Bonnans et al., 2014; Yang et al., 2014; Kumar et al., 2017). While much is known about cell signaling and functional consequences in response to biochemical cues, such as nutrients, growth factors, and hormones, the impacts of biophysical modulations on tissue growth and homeostasis in health and diseases are understudied. With developments in advanced engineering substrates and apparatus to better mimic mechanics of microenvironments in native tissues, biophysical factors, such as matrix stiffness, cell-cell contacts, and local hemodynamic forces, have been appreciated for their roles in regulating cellular functions and phenotypes (Lampi and Reinhart-King, 2018; Mohammadi and Sahai, 2018; Maurer and Lammerding, 2019; Sheetz, 2019; Yang et al., 2019). However, our knowledge is rather limited for how cells sense the changes of microenvironments and transduce such mechanical stimuli to biochemical signaling cascades governing cellular responses.

The Hippo pathway, since it was identified in *Drosophila melanogaster* less than two decades ago (Harvey et al., 2003; Pantalacci et al., 2003; Udan et al., 2003; Wu et al., 2003), has been extensively studied and now regarded as a master regulator of organ development, regeneration, and carcinogenesis, *via* integrating extrinsic and intrinsic cues that reshape cellular transcription programs (Pan, 2010; Meng et al., 2016; Moya and Halder, 2019; Dey et al., 2020). The core of the mammalian Hippo pathway is a kinase cascade consisting of the Mammalian STE20-like kinase 1/2 (MST1/2) and the Large tumor suppressor kinase 1/2 (LATS1/2). When cells are exposed to growth-inhibiting signals, MST1/2 phosphorylate LATS1/2 at their hydrophobic motif and thus activate LATS1/2, which in turn phosphorylate and inactivate two transcription factors Yes-associated protein (YAP) and Transcriptional coactivator with PDZ-binding motif (TAZ) by inducing their cytoplasmic retention and protein degradation. When the Hippo pathway is inactivated by growth-promoting signals, YAP and TAZ are dephosphorylated and thus located in the nucleus, where they bind to the TEAD family of transcription factors and drive transcription programs that promote cell proliferation, mobility, and stemness. In addition, novel Hippo kinases (e.g., MAP4Ks), accessory proteins (e.g., SAV1, NF2, and MOB1/2), and tuning machineries (e.g., the STRIPAK-PP2A complex) have been shown as indispensable components, among dozens of many other peripheral regulators that finely tune the pathway (Misra and Irvine, 2018; Ma et al., 2019; Zheng and Pan, 2019), for relaying environmental signals to the core of the Hippo pathway.

**FIGURE 1:**
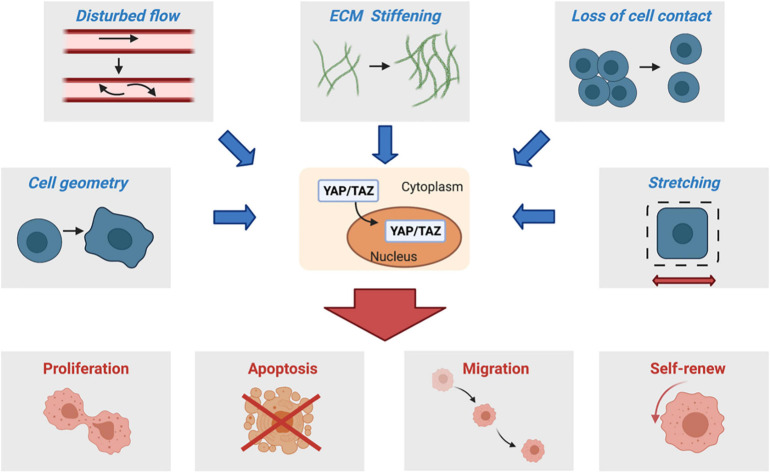
Changes in the biophysical environments, such as those resulting from ECM stiffening, loss of cell contact, disturbed flow, cell spreading, and stretching, promote nuclear translocation of YAP/TAZ and thus activate their transcription programs for cell proliferation, survival, migration, and self-renew, among others.

There have been many comprehensive reviews on the Hippo pathway regulation and its biological roles as cited above. In this review, we will focus on the molecular mechanisms by which the Hippo pathway is modulated by mechanical cues in microenvironments (Figure 1). Furthermore, we will summarize the recent progress on how mechanoregulation of the Hippo pathway contributes to human disease development.

## General Roles of Mechanical Signals in Cell Behaviors and Fate Decision

Cells and tissues perceive microenvironmental physical forces that are generated both internally and externally. Internal forces are mainly generated by cytoskeleton dynamics (endogenous forces), while external forces result from both environment and neighbor cells (applied forces) (Chen, 2008). The resulting mechanical cues, including tissue stiffness, stretch, and shear stress, modulate cell behaviors, such as cell proliferation, spreading, migration, and differentiation. The process through which biophysical forces are sensed and converted into biochemical signals that elicit responses is termed *mechanotransduction* (Ingber, 2006; Dasgupta and McCollum, 2019). This process was typically divided into mechanosensing (the act of sensing a mechanical stimulus), mechanotransmission (the act of transmitting such a stimulus to signaling events), and mechanoresponse (the functional response of cells to the mechanical stimulus) (Hoffman et al., 2011).

Mechanosensing is usually initiated at the cell surface, where plasma membrane receptors, their associated proteins, and the plasma membrane itself sense and propagate mechanical cues to trigger signaling cascades that generate mechanoresponses. Integrins, G protein-coupled receptors, Enzyme-linked Receptors (i.e., Receptor Tyrosine Kinase), and Ion Channels can be all such “mechanosensors” (Chen et al., 2017). Forces applied to these receptors or on/through their ligands change configurations of the receptors, leading to enzymatic reactions and/or cytoskeleton remodeling. Our understanding of membrane-associated mechanosensors has been greatly improved in the past decade. For example, Integrins and Piezo channels, evolutionarily conserved in mammalian cells, are mechanosensitive, characterized by the activation of corresponding downstream effectors and regulation of their biological functions in response to mechanical stimuli (Kechagia et al., 2019; Xiao, 2020). Besides the membrane-associated receptors, mechanical forces can be directly transmitted to the nucleus, the envelope of which responds to mechanical compression or stretch to alter transport of transcription factors and other nucleus-cytoplasm shuttling proteins, as well as remodel nucleoskeleton (Dahl et al., 2008; Wang et al., 2009; Enyedi et al., 2016; Elosegui-Artola et al., 2017; Hoffman et al., 2020).

It was recognized a long time ago that mechanical signals during development dynamically control gene transcription programs to determine cell fate and organ growth (Discher et al., 2005; Engler et al., 2006). However, it was then unclear how these mechano-responsive transcription programs are regulated by the mechanotransduction initiated by membrane proteins and cytoskeletons. Emerging efforts have been made to elucidate the signaling cascades that link membrane mechanosensors to the nuclear transcription machinery. One such “missing” signaling cascade that has been increasingly appreciated is the Hippo pathway, though many details of mechanoregulation of this pathway remain yet to be determined.

## YAP and TAZ Are at the Center Stage of Mechanotransduction

**FIGURE 2:**
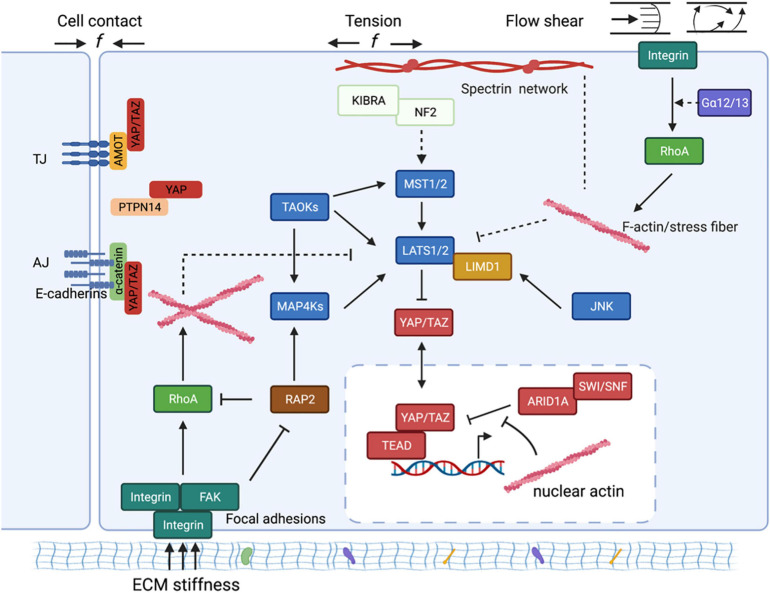
The schematic diagram of molecular mechanisms by which mechanical cues regulate YAP/TAZ. Mechanical cues, such as cell-cell contact, ECM stiffness, externally applied mechanical stretch, and flow shear, control YAP/TAZ activity through both Hippo-dependent and -independent pathways. The core kinase cascade consisting of MST1/2 and LATS1/2, as well as novel Hippo kinases such as MAP4Ks, respond to the mechanical cues to modulate phosphorylation and localization of YAP/TAZ. Mechanical cues can also bypass these kinases and act through both cytoplasmic and nuclear actins to modulate YAP/TAZ localization. (1) In response to cell-cell contact, AMOT directly binds to YAP and thus sequesters YAP at tight junctions regardless of YAP phosphorylation status. AMOT can simultaneously act through NF2/Merlin to activate the MST1/2-LATS1/2 kinase cascade to induce phosphorylation and inactivation of YAP. Adherens junction (AJ) protein E-cadherin, upon cell confluence, *trans*-dimerize and subsequently inactivate YAP/TAZ through the MST1/2-LATS1/2 kinase cascade. Cell-cell contact inhibition also promotes direct interaction between PTPN14 and YAP, and thus leads to cytoplasmic translocation of YAP. (2) ECM stiffness sensed by focal adhesion promotes actin polymerization and stress fiber formation. The Hippo kinase cascade is inactivated by actin polymerization and stress fiber formation at high ECM stiffness. On the other hand, polymerized nuclear actin, under high ECM stiffness, binds to ARID1A-SWI/SNF complex, subsequently relieving its sequestration of YAP/TAZ. In addition, the stiffness-regulated GTPase RAP2 directly activates MAP4K4/6/7 as well as inhibits Rho GTPases, leading to LATS kinase activation and YAP/TAZ inhibition. Stiffness-activated JNK can phosphorylate LIMD1, which directly binds to LATS1/2 and reduces their kinases activities, thus activating YAP/TAZ. (3) Mechanical stretch or tension also acts through actin cytoskeleton to modulate YAP/TZ activities. Spectrin, a cytoskeletal protein, serves as a key linker that connects the cellular tension-sensing system to the Hippo regulation network. (4) Flow shear patterns and speeds differentially regulate activity of the Hippo kinase cascade in endothelial cells *via* an integrin–Gα12/13–RhoA axis.

Two landmark studies from the Piccolo group and the Sasaki group (Dupont et al., 2011; Wada et al., 2011) published in 2011 opened a new avenue for us to understand how mechanical cues modulate the activities of nuclear factors that dictate transcription programs to control cell behaviors and fate. Their studies demonstrated for the first time that YAP and TAZ, the Hippo pathway effectors, are mechanotransducers to relay cytoskeletal tension to nuclei and to regulate cell functions, and more importantly, they are functionally indispensable for the biological outputs of mechanical cues. Thereafter, new nuclear factors (i.e., β-catenin, Twist) and new mechanosensitive signaling molecules, such as RAP2 and MAP4K, have been identified (Benham-Pyle et al., 2015; Wei et al., 2015; Li et al., 2018; Meng et al., 2018), thus greatly advancing our mechanistic understanding of mechanotransduction and the Hippo pathway regulation. In the following session, we will summarize the most updated knowledge of how each type of mechanical cues regulates YAP and TAZ and the biological context(s) for the corresponding regulation (Figure 2).

### Extracellular Matrix (ECM) Stiffness

The mechanical microenvironment of cells is largely determined by their neighbor cells and surrounding extracellular matrix (Discher et al., 2005; Paszek et al., 2005; Engler et al., 2006; Wells, 2008). The difference in ECM composition leads to unique three-dimensional networks in various tissues and determines the rigidity or elasticity of substrates where cells habituate and grow. This physical characteristic of the microenvironment profoundly influences cell phenotypes, not only their morphology but also internal cytoskeleton organization and trafficking (Myers et al., 2011; Wong et al., 2020). To investigate the effect of ECM stiffness on cell functions, engineered hydrogels interfacing with ECM proteins, such as fibronectin and collagens, have widely been used as substrates to grow cells *in vitro*. As a result, ECM stiffness has been shown to regulate cell growth, migration, and differentiation, among many other important cell behaviors.

It was first shown by the Piccolo group that increased ECM stiffness promotes nuclear localization of YAP and TAZ and upregulation of their target genes (Dupont et al., 2011). Though the underlying molecular mechanisms were not fully uncovered then, they demonstrated that actin cytoskeleton tension resulting from manipulations of cell spreading and substrate rigidity constitutes the key link between ECM stiffness and YAP/TAZ activation. High ECM stiffness promotes cell spreading and subsequently leads to actin cytoskeleton tension, which in turn results in nuclear translocation of YAP/TAZ with a to-be-defined mechanism. One key player that relays stiffness signals to YAP/TAZ is RhoA GTPase, which can sense ECM stiffness through focal adhesion and in turn promote actin polymerization and stress fiber formation. Actin cytoskeleton can regulate YAP and TAZ through both Hippo-dependent and -independent pathways (Dupont et al., 2011; Wada et al., 2011). On one hand, the Hippo pathway can be inactivated by actin polymerization and stress fiber formation at high ECM stiffness, and activated by relaxed actin cytoskeletons at low ECM stiffness. Phosphorylation and localization of YAP/TAZ are thus modulated by ECM stiffness through the Hippo pathway (Codelia et al., 2014; Li et al., 2018; Meng et al., 2018). On the other hand, the ARID1A-SWI/SNF complex binds to YAP/TAZ and thus prevents the interaction between YAP/TAZ and TEAD. Polymerized nuclear actin, under high ECM stiffness, binds to ARID1A-SWI/SNF complex, subsequently relieving its sequestration of YAP/TAZ (Chang et al., 2018).

To date, our mechanistic understanding of how ECM stiffness regulates YAP/TAZ remains incomplete as emerging players have been reported. ECM stiffness can be sensed by cells through focal adhesions, as cells use integrin to pull integrin ligands in the ECM. In addition to common integrin ligands such as collagens and fibronectin, an ECM proteoglycan, Agrin, also act as an extracellular mediator of matrix mechanotransduction to regulate YAP/TAZ *via* integrins, its specific membrane receptor LRP4, and the actin cytoskeleton (Chakraborty et al., 2017; Chakraborty et al., 2020). Similarly, matricellular protein thrombospondin-1, acts through integrin-1 and the Hippo pathway, but not actin cytoskeleton, to activate YAP/TAZ (Yamashiro et al., 2020). Besides signaling through integrin/focal adhesion, plasma membrane protein Caveolin-1 (CAV-1) also play a critical role in mechanoregulation of YAP/TAZ (Moreno-Vicente et al., 2018). CAV1 loss promotes the interaction between YAP and the 14-3-3 protein YWHAH in an F-actin-dependent but Hippo-independent manner.

Besides membrane proteins, signaling transducers like small GTPases and protein kinases also mediate stiffness signals to the Hippo pathway. Small GTPases, such as RhoA and RAC1, have been demonstrated for their roles as molecular switches for stiffness sensing (Bae et al., 2014). Another GTPase, RAP2, also senses stiffness cues from focal adhesions (Meng et al., 2018; Yamashiro et al., 2020), as ECM stiffness modulates the GDP/GTP loading status of RAP2. Active/GTP-loading RAP2 activates non-canonical Hippo kinases MAP4K4/6/7 by direct binding to their hydrophobic motif leading to LATS activation and YAP/TAZ inhibition. JNK is also an important mediator of stiffness sensing for YAP/TAZ regulation in epithelial cells (Codelia et al., 2014). Stiffness-activated JNK can phosphorylate LIMD1, which directly binds to LATS1/2 and reduces their kinases activities, eventually leading to activation of YAP/TAZ.

### Static and Cyclic Stretch

Almost all adherent cells, *in vitro* or *in vivo*, experience physical forces transmitted between cells and across tissues, and one of the most common forces in animals is tension caused by mechanical stretch. For example, when cells undergo tissue growth or disease progression, the increase in ECM stiffness generates a static tension that stretches cells. Such kind of stretch forces, as previously discussed, modulate cell behaviors *via* a YAP/TAZ-dependent mechanism. In addition to ECM stiffening, stretching forces from external sources, such as tissue mechanical strain and artificial cell stretching devices, are able to regulate cell functions through the interplay of the Hippo pathway (Driscoll et al., 2015; Fletcher et al., 2018). Moreover, recent research has linked YAP dysregulation to malfunction of the cardiovascular system caused by pressure overload (Byun et al., 2019), suggesting a critical regulatory role of the Hippo-YAP/TAZ pathway in cells in response to various magnitudes of stretch force.

Static stretch activates YAP/TAZ by suppressing capping and severing proteins of F-actins (Aragona et al., 2013). Additionally, the stretch force applied to the nucleus may decrease the mechanical restriction of nuclear pores, thus facilitating nuclear transport of YAP/TAZ (Elosegui-Artola et al., 2017). The linker of nucleoskeleton and cytoskeleton (LINC) complex, which transfers cytoskeletal strain to the nucleus, is a key player for mechanical stretch to activate YAP/TAZ in this process (Driscoll et al., 2015; Koushki et al., 2020).

Cyclic stretch has also been reported to activate YAP/TAZ by suppression of the Hippo pathway (Codelia et al., 2014), even in cells on soft matrices (Cui et al., 2015). In Drosophila, the physiological mechanical strain can drive activation and nuclear localization of YAP/TAZ homolog Yki in *Drosophila* follicular epithelium to promote cell proliferation, *via* inactivating the LATS1/2 homolog Warts (Fletcher et al., 2018). The novel Hippo kinase Msn plays a critical role in sensing mechanical stretch in *Drosophila* gut. Mechanical stretch dissociates Msn from the plasma membrane and thus prevents phosphorylation and activation of Msn by the Tao kinase, which in turn activates expansion of *Drosphila* gut stem cells and leads to intestinal hyperproliferation (Li et al., 2014, 2018).

In mammals, cyclic stretch is known to act through thrombospondin-1/RAP2 in blood vessel cells to activate YAP and promote vascular remodeling (Yamashiro et al., 2020). Thrombospondin-1 acts *via* integrin αvβ1 to form focal adhesions and promotes nuclear shuttling of YAP by inactivating the RAP2 GTPase, results in vascular remodeling in response to the pulsatile blood flow and pressure. It is worth mentioning that externally applied mechanical stretch and ECM stiffening shared many mechanosensing machineries, such as RAP2 and Msn/MAP4Ks, to regulate YAP/TAZ activities. It would be important to understand how these universal machineries work in concert with cell-specific mechanosensors (e.g., VEGFR2 in endothelial cells) to control mechanoresponses of cells in future.

### Cell-Cell Contact

Cell-cell contact inhibition is the phenomenon that cells avoid proliferating as they achieve convergence in monolayers. During carcinogenesis, transformed cells slowly lose this character (Hanahan and Weinberg, 2011). Loss of contact inhibition allows the transformed cells to overcome physical restraint and in many cases contributes to cancer cell aggressiveness. Contact-dependent signaling is one of the first extracellular stimuli discovered to regulate the Hippo pathway ([Bibr B129][Bibr B130]). Direct contact between normal cells activates the Hippo kinases, thus leading to phosphorylation and cytoplasmic retention of YAP/TAZ, triggering cell cycle arrest and/or autophagy (Pavel et al., 2018). In contrast, hypophosphorylation and nuclear localization of YAP/TAZ have been associated with loss of contact inhibition of cancer cells resulting from somatic mutations (Zhao et al., 2007; Zhang et al., 2010; Tranchant et al., 2017; Frank et al., 2018; Ouyang et al., 2020).

Various mechanisms have been proposed to explain how cell-cell contact inhibition modulates activities and localization of YAP/TAZ. These mechanisms can be roughly grouped into two types. The first one usually involves cis-interaction of cell membrane proteins at high confluence. For example, for tight junctions (TJs) form between cells, angiomotin (AMOT) complex at TJs is activated and transmit the antiproliferative signal from TJs to YAP via two independent mechanisms: AMOT can directly bind to YAP and thus sequester YAP at TJs, and/or AMOT can activate Merlin/NF2 to trigger LATS1/2-dependent YAP phosphorylation (Li et al., 2015). In addition to TJs, adherens junctions (AJs) protein E-cadherin, in confluent cells, *trans*-dimerize and subsequently stimulate MST1/2-LATS1/2 kinase cascade to inhibit activities of YAP/TAZ (Kim et al., 2011). PTPN14, a protein tyrosine phosphatase, can inhibit YAP transactivation activity through a direct interaction in response to cell confluence (Wang et al., 2012; Liu et al., 2013). The other type of mechanisms involves cell geometry and actin cytoskeleton remodeling. High cell density, as well as low ECM stiffness, reduces adhesive area and alters cell shape, which leads to inactivation of RhoA and subsequent reduction of stress fiber of actin cytoskeleton, which can inactivate YAP/TAZ through both Hippo kinases-dependent and -independent mechanisms (Aragona et al., 2013; Meng et al., 2018; Chang et al., 2018). Spectrin has been recognized as a key cytoskeletal protein that restricts YAP/TAZ activity in response to mechanical cues, such as cell-cell contact inhibition, through remodeling actin cytoskeleton particularly at cortical areas of cells. Loss of Spectrin proteins results in hyperactivation of YAP/TAZ, likely by elevating cortical myosin II activity, leading to cell over-proliferation even when cells confluence is reached in both mammals and *Drosophila* (Deng et al., 2015; Fletcher et al., 2015; Wong et al., 2015; Deng et al., 2020).

### Fluid Shear Stress

Shear stress, a fluid frictional force, is another major mechanical stimulus maintaining tissue homeostasis. Indeed, one of the most studied cell types in mechanotransduction is vascular endothelial cells (ECs), lining in the innermost layer of blood vessels. ECs are constantly subjected to shear stress from blood flow, and it is well recognized that ECs are able to sense and respond to changes in flow direction, pulsatility, and magnitude of shear stress *via* mechanosensors and mechanosensitive signaling pathways. As a result, endothelial phenotypes are highly associated with local blood flow patterns and distinct in different regions of the vascular tree. Three recent studies independently confirmed that flow patterns modulate endothelial phenotypes through regulation of YAP/TAZ activities: unidirectional laminar flow suppresses YAP/TAZ activities to keep ECs quiescent and inert to inflammatory cells, while disturbed oscillatory flow activates YAP/TAZ to promote a pro-proliferative and -inflammatory EC phenotype (Wang K. et al., 2016; Wang L. et al., 2016; Xu et al., 2016).

Mechanistically, the integrin–Gα13–RhoA axis was first reported to mediate the flow regulation of YAP/TAZ activities in ECs (Wang L. et al., 2016) and two recent studies revealed that disturbed flow acts through integrin α5β1 to induce YAP nuclear translocation and promote the pro-atherogenic responses, via c-Abl kinase and phosphodiesterase 4D5, respectively (Li et al., 2019; Yun et al., 2019). However, the mechanisms regarding how the flow-activated integrin signaling cascades crosstalk with Hippo kinases to modulate YAP/TAZ activities remain to be studied. In addition to the integrin-mediated mechanisms, it has been shown that short-term unidirectional laminar flow (15 dyne/cm^2^ for 10 min) increases the nuclear localization of YAP in a LATS1/2-independent but an angiomotin-regulated manner (Nakajima et al., 2017). Last but not the least, caveolae, the plasma membrane microdomain, is known to sense shear stress signals and have been shown to relay such mechanical cues through the Hippo pathway to facilitate mechanoregulation of YAP/TAZ (Rausch et al., 2019). However, whether the caveolae-dependent mechanism mediates the flow regulation of endothelial phenotypes remains to be determined.

In addition to ECs, many other cell types, such as metastatic tumor cells and mesenchymal stem cells, are known to perceive shear stress stimuli and transduce the resulting biochemical signals in regulating cellular functions (Lee et al., 2017, 2018; Qin et al., 2019). In fact, the connection between shear stress and YAP was first reported in mesenchymal stem cells, where it was shown that exposure of mesenchymal stem cells to shear stress enhances YAP expression to promote their differentiation into chondrocytes (Zhong et al., 2013). More *in vitro* and *in vivo* studies are warranted to validate the roles of YAP/TAZ as mechanotransducers in modulating biological functions in the above-mentioned cell types.

## Mechanoregulation of YAP/TAZ in Human Diseases

Mechanoregulation of YAP/TAZ plays a critical role in normal development and aging processes. For instance, it is known that physiological substrate stiffness directs human pluripotent stem cell specification by influencing cytoskeleton arrangement and intracellular tension through the YAP-TEAD complex (Pagliari et al., 2021). The behavior of limbal epithelial stem cells is strongly influenced by changes in corneal substrate stiffness, *via* the activation of YAP-dependent mechanotransduction pathways (Gouveia et al., 2019). Furthermore, an age-related stiffness drives YAP/TAZ-mediated pathogenic expression of ECM proteins, ultimately disrupting muscle stem cell fate (Stearns-Reider et al., 2017).

**FIGURE 3:**
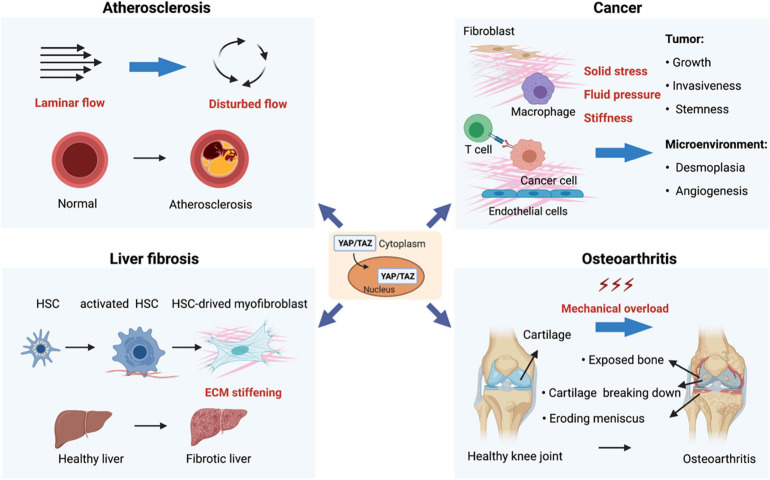
Mechanoregulation of YAP/TAZ in human diseases. (1) Disturbed flow-activated YAP/TAZ is a key factor that promotes atherogenesis. (2) Mechanical properties in the tumor microenvironment (such as solid stress, fluid pressure, and stiffness) act through YAP/TAZ to regulate various aspects of tumor initiation and progression. (3) YAP/TAZ-mediated mechanoresponses strongly promote organ fibrosis. For instance, hepatic injuries and subsequent inflammatory responses activate quiescent hepatic stellate cells (HSCs), leading to activation and expansion of HSCs and accumulation of extracellular. (4) Osteoarthritis (OA) is mainly caused by mechanical overload, and YAP is both necessary and adequate to preserve cartilage homeostasis in OA.

Emerging evidence connects YAP/TAZ dysregulation by mechanical cues to various human diseases. Although the underlying mechanisms remain to be defined in many cases, understanding of the Hippo pathway dysregulation by mechanical cues in human diseases potentially can provide us insights into new therapeutic strategies for these diseases (Figure 3).

### Cardiovascular Diseases (CVDs)

The cardiovascular system is subjected to continuously shifting mechanical signals, including stretch, compression, distortion, and shear. Mechanotransduction profoundly influences the development of the cardiovascular system and the regulation of physiological functions (Garoffolo and Pesce, 2019). The roles of YAP/TAZ in CVDs have been well summarized in a recent review (Yu et al., 2020). Aberrant activation of YAP/TAZ contributes to a variety of cardiovascular conditions: atherosclerosis, pulmonary hypertension, myocardial hypertrophy, angiogenesis, restenosis, and myocardial fibrosis, while hypoactivation of YAP/TAZ is associated with aortic aneurysms, aortic dissection, reperfusion of myocardial ischemia, and myocardial infarction. Our review will solely focus on YAP/TAZ dysregulation in mechanical cues resulting from/in pulmonary hypertension, atherosclerosis, and cardiac hypertrophy.

#### Pulmonary Hypertension (PH)

Pulmonary hypertension (PH) refers to a pathophysiologic condition of increased blood pressure within the arteries of the lungs with many possible causes (Hoeper et al., 2013).

One feature of PH is remodeling of small pulmonary arteries caused by hyperproliferation of vascular smooth muscle cells (VSMCs) and myofibroblasts, leading to aberrant deposition of collagen and elastin and vascular ECM stiffening. A number of cross-sectional studies suggest that YAP/TAZ activation downstream of ECM stiffening is a key driver of PH (Bertero et al., 2015b, 2016). Vascular remodeling and stiffening activate YAP/TAZ, which then drive a transcription program that promotes ECM deposition and crosslinking and further enhances vascular remodeling and stiffening, thus constituting a forward feedback loop. Specifically, ECM stiffening activates YAP/TAZ in myofibroblasts, endothelial cells, and VSMCs and thus facilitates proliferation of these cells. Furthermore, YAP/TAZ in these cells activate genes involved in ECM synthesis (i.e., collagens) and crosslinking (i.e., lysyl oxidase) (Bertero et al., 2015b). In addition, YAP/TAZ also link mechanical stimuli to dysregulated vascular metabolism associated with PH. ECM remodeling controls the expression of glutaminase by activating YAP/TAZ, leading to activation of glutaminolysis and anaplerosis, fostering PAECs and PASMCs proliferation and migration. In mouse models, LOX inhibitors dampened nuclear YAP/TAZ and improved end-stage manifestations of PH (Bertero et al., 2016). Increased pulsatility and shear stress have been associated with YAP/TAZ activation in pulmonary vascular ECM remodeling and pulmonary adventitial myofibroblast proliferation. It is unknown, however, whether mechanical signals from increased pulsatility and shear stress alone will be sufficient to activate YAP/TAZ in the adventitial myofibroblasts in the absence of a rigid matrix (Thenappan et al., 2018).

#### Atherosclerosis

Atherosclerotic plaques develop preferentially at arterial bifurcations and high curvatures but not straight segments of arteries. The site-specific manner of lesion formation suggests that local hemodynamic forces acting on vessel walls play a critical role in exerting atheroprone or atheroprotective effects on vascular cells. Indeed, as discussed previously, disturbed flow, but not steady laminar flow, activates endothelial YAP/TAZ in ECs, leading to upregulations of pro-atherogenic (e.g., *VCAM1*) and YAP/TAZ-target genes (e.g., *CTGF* and *CYR61*) *in vitro* (Wang K. et al., 2016; Wang L. et al., 2016; Xu et al., 2016). In agreement with the *in vitro* findings, a higher level of YAP/TAZ activation was detected in atheroprone regions than in atheroprotective regions mouse aorta, suggesting the involvement of disturbed flow-activated YAP/TAZ in atherogenesis. In ApoE^–/–^ mice, knockdown of YAP/TAZ expression decreases the pro-atherogenic phenotypes of vascular cells and attenuates lesion development in atheroprone regions of arteries. On the other hand, atherosclerosis is encouraged by overexpression of endothelial YAP, or systemic TAZ, or a constitutively active YAP/TAZ mutation (Wang L. et al., 2016; Li et al., 2019). Taken together, these studies reassuringly uncovered the importance of YAP/TAZ as mechanotransducers in vascular cells and atherogenesis.

Besides YAP/TAZ, hemodynamic forces activate a number of signaling pathways to regulate endothelial phenotypes associated with atherosclerosis, *via* other transcription factors, such as KLF2, KLF4, NRF2, HIF-1α, NF-κB, AP-1, and a few others (Niu et al., 2019). These transcription factors, collectively referred to mechanosensitive transcription factors (MSTFs), crosstalk with one another to regulate ECs upon exposure to hemodynamic forces, as well as control cellular responses to oxidative stress, inflammation, and metabolic programming. Understanding how YAP/TAZ work in concert with these MSTFs in endothelium homeostasis will be important for us to obtain a panoramic view of the mechanosensing signaling network in endothelial cells.

In addition to their roles in ECs, the phenotypic switch of VSMCs from contractile to synthetic phase is regulated by YAP/TAZ activities, in response to stretch or wall stress-induced vascular remodeling (Wang et al., 2018). Stretching VSMCs activates their PI3K-PDK1 signaling, which then prevents the Hippo kinase cascade from inactivating YAP/TAZ.

#### Cardiac Hypertrophy

Cardiac hypertrophy is an adaptive response to hemodynamic overload. In the beginning, cardiac hypertrophy is beneficial because it increases the number of contractile units and reduces the ventricular wall pressure to a normal level according to Laplace’s law. However, as the adjustment is physically limited, cardiac hypertrophy will lead to heart failure (Nakamura and Sadoshima, 2018).

Experimental studies have supported the concept that mechanical cues, such as hemodynamic overload, predominantly affect cardiomyocytes (CMs) by stretching. Integrins and the cytoskeleton or sarcolemmal proteins (e.g., phospholipases, ion channels, and ion exchangers) are two main types of mechanosensors, by which hemodynamic overload is coupled to intracellular signals responsible for the hypertrophic response (Ruwhof and van der Laarse, 2000).

Hemodynamic overload includes two forms: pressure overload and volume overload. Endogenous YAP is a crucial mediator of hypertrophy in response to volume overload, which regulates CM growth and survival in the adult mouse myocardium in response to myocardial ischemic injury (Del Re et al., 2013). It was believed that compensatory cardiomyocyte hypertrophy is at least partially regulated by Yap1 after chronic myocardial infarction and a Yap1 deficit impairs the growth response to stress in heart, contributing to worsened operation. In response to pressure overload, endogenous YAP is triggered in CMs through a RhoA-dependent mechanism. Heterozygous YAP depletion inhibited hypertrophy, but increased fibrosis and apoptosis, and decreased cardiac functions (Byun et al., 2019). These findings highlight YAP as a potential target for myocardial infarction and hypertension.

### Organ Fibrosis

Inflammation and wound-healing process following tissue injury and/or an idiopathic disease induce profibrotic responses, including abnormal ECM synthesis and deposition by fibroblasts and tissue stiffening and thickening. Such changes in structural and mechanical properties of the tissue are usually irreversible and lead to the formation of scar tissue, also known as fibrosis. The physical features of fibrotic tissues, together with the unresolved inflammation, continuously stimulate fibroblasts, resulting in pathological accumulation of ECM components and permanent tissue damage (Rockey et al., 2015; Tschumperlin et al., 2018).

YAP/TAZ serve as critical mechanotransducers in fibroblasts, coordinating profibrotic responses in various tissues, such as hepatic fibrosis, pulmonary fibrosis, kidney fibrosis, cardiovascular fibrosis, and others (Liu et al., 2015; Kim et al., 2019). The initial increase in ECM stiffness activates YAP/TAZ in fibroblasts, encouraging the development of profibrotic mediators and excessive deposition of ECM components. This results in progressive tissue stiffening, thereby forming an activation feed-forward loop to promote tissue fibrosis (Bertero et al., 2015a; Liu et al., 2015). Moreover, YAP/TAZ act as a molecular link between fibrosis and cancer. Fibrotic ECM stimulates cell proliferation and changes cell polarity, thereby promoting tumor development and growth (Noguchi et al., 2018). Various studies have assessed the efficacy of inhibiting YAP/TAZ activity as a new therapeutic strategy to reverse fibrosis (Liang et al., 2017; Haak et al., 2019; Alsamman et al., 2020; Dey et al., 2020), and the results of those have unfolded a promise of YAP/TAZ-targeting therapies for organ fibrosis.

### Musculoskeletal Disorders (MSDs)

MSDs refers to diseases that affect the muscles, bones, and joints, which mainly tendinitis, carpal tunnel syndrome, osteoarthritis (OA), rheumatoid arthritis (RA), etc.

Mechanical load has been shown to activate YAP by increasing the expression of nuclear accumulation of YAP. Hyperactivation of YAP by sustained mechanical overload or YAP overexpression alone is sufficient to induce skeletal muscle hypertrophy (Goodman et al., 2015; Iyer et al., 2019; Owens et al., 2020). Besides responding to mechanical load, YAP/TAZ play a crucial role in muscle cell stemness and myogenesis (Figeac et al., 2019; Zhang L. et al., 2019). However, more studies are required as their atrophic roles of YAP/TAZ in muscle cells have also been reported (Gnimassou et al., 2017), and more details about the connection between mechanical stimuli and YAP/TAZ signaling would further improve our understanding of the roles YAP/TAZ in mechanotransduction and muscle homeostasis.

OA is mainly caused by mechanical overload (Glyn-Jones et al., 2015), and it has been reported that YAP is both necessary and adequate to preserve cartilage homeostasis in OA (Deng et al., 2018; Zhang Q. et al., 2019). A few recent studies demonstrated that suppressing YAP activity is effective in attenuating OA progression (Gong et al., 2019; Thorup et al., 2020; Zhang et al., 2020). However, research is still lacking on how the mechanical forces, especially compression and hydrostatic pressure, regulate Hippo-YAP/TAZ pathway in chondrocytes in healthy cartilage and during OA development.

### Cancer

The biophysical factors in the tumor microenvironment play a pivotal role in disease progression and treatment outcomes. Common physical features that hinder cancer treatment, including solid stresses, interstitial fluid pressure, stiffness, and tumor microarchitecture, have been nicely reviewed by Nia HT, Munn LL, and Jain RK very recently (Nia et al., 2020). It has long been known that the changes in tumor microenvironment trigger signaling pathways to fuel cancer progression, immune blockade, and cancer resistance to therapy. Among the mechanosensitive pathways, the dysregulation of the Hippo-YAP signaling plays a central role in tumor development as it has been connected to most of the physical features in tumors. Emerging efforts have been made to elucidate the molecular mechanisms by which mechanical properties of various tumor types act through YAP/TAZ to promote cancer pathology.

Loss of cell-cell contact inhibition and unchecked cell growth are hallmarks of cancer, and the Hippo pathway is known to mediate contact inhibition and cell proliferation. Indeed, dysregulation of the Hippo signaling and hyperactivation of YAP/TAZ not only abolish the cell cycle regulation, as a result of losing contact inhibition, but also promote the transformation of mammary epithelial cells (Zhao et al., 2007, 2008). As the tumor grows, the changes in mechanical properties of the microenvironment continuously contribute to cancer progression and malignancy. Numerous studies have documented that stiff substrates activate YAP/TAZ to increase primary cancer cell growth, migration, and chemotherapy resistance (Lin et al., 2015; Chakraborty et al., 2017; Foster et al., 2017; Meng et al., 2018; Santinon et al., 2018; Molina et al., 2019; Ghasemi et al., 2020; Liu et al., 2020; Qin et al., 2020). Furthermore, it has been shown that Ras signaling-mediated oncogenic transformation of normal cells requires a stiff and/or fibrotic microenvironment (Meng et al., 2018; Panciera et al., 2020).

Mechanistically, the ECM-activated YAP/TAZ regulates the expression of various cytoskeletal regulators in cancer-associated fibroblasts and increases intracellular isometric tension, thus forming a forward feedback loop to enhance the tumor microenvironmental rigidity and support cancer cell growth and invasion (Calvo et al., 2013; Foster et al., 2017). It is speculated that YAP/TAZ hyperactivation is required for cancer cells to overdrive the mechanical checkpoints for growth (Aragona et al., 2013). In addition to cancer cells and cancer-associated fibroblasts, ECM stiffening during tumor progression induces vascular cell growth and allows blood vessel infiltration, potentially through the Agrin-YAP-dependent mechanism (Chakraborty et al., 2020). Indeed, it has recently been shown that the increases in tissue rigidity at metastatic sites enhance cancer angiogenesis and elevate their resistance to anti-angiogenesis therapy (Shen et al., 2020).

Cancer cells migrate and metastasize in blood and lymphatic vessels. Therefore, it would be important to know the flow regulation of YAP/TAZ in cancer cells. It has been reported that fluid shear stress induces YAP/TAZ activation to promote cancer cell migration and proliferation (Lee et al., 2017, 2018; Qin et al., 2019), suggesting a causative role of YAP/TAZ activation in cancer metastasis. In this regard, modulation of Hippo-YAP/TAZ signaling may represent a novel strategy for anti-metastasis therapies. In addition, the role of YAP/TAZ-mediated mechanosensing in tumorigenesis may be more complex and multi-functions in context-dependent manners and therefore worth further exploring. For example, a recent study in mice showed that activation of the YAP/TAZ in peritumoral normal hepatocytes suppressed liver tumor growth *via* cell competition mechanism against tumor cells (Moya et al., 2019). It would be important to elucidate the exact roles of YAP-dependent mechanoresponses in physical interaction between normal and tumor cells in future studies.

## Conclusion

Research in the last decade has established an indispensable role of the Hippo-YAP pathway in mechanobiology of tissue growth and homeostasis, although more research is still needed to finely define biological roles and molecular mechanisms for specific pathogenesis processes. Many studies have implicated that the Hippo pathway may serve as a signaling integration hub through interpreting both mechanical and biochemical cues. One recent study worth particularly mentioning is from Barry Thompson group. They reported that mechanically induced Yki nuclear shuttling requires growth factors-activated PI3K-AKT signaling in *Drosophila*. This coordination of the force-regulated Yki signaling and the PI3K-AKT signaling couples cell polarity and tissue mechanics to nutritional cues in tissue growth control (Borreguero-Muñoz et al., 2019). Future investigation into the crosstalk between YAP-mediated mechanotransduction and biochemical cues in the disease microenvironment will be crucial for us to obtain a more comprehensive knowledge of pathogenesis associated with dysregulated tissue mechanics. Moreover, it will be also important to understand how the Hippo-YAP pathway works in concert with other mechanosensing mechanisms (e.g., KLFs, MRTF-SRF, TWIST, and β-catenin) to generate a singular but context-specific mechanoresponse in cells. Furthermore, as industrial and academic efforts are emerging for developing YAP-targeting agents, testing such agents in animal models of diseases associated with mechano-dysregulation will likely lead to novel therapies.

## Author Contributions

XC, K-CW, and ZM worked together to conceive and draft the manuscript. All authors contributed to the article and approved the submitted version.

## Conflict of Interest

The authors declare that the research was conducted in the absence of any commercial or financial relationships that could be construed as a potential conflict of interest.
